# The 6th of February earthquake and the Turkish Society of Pediatric Nephrology—organizational aspects of pediatric kidney care

**DOI:** 10.1093/ndt/gfad138

**Published:** 2023-07-03

**Authors:** Sevcan A Bakkaloğlu, Önder Yavaşcan, Alev Yılmaz, Kaan Gülleroğlu, Belde Kasap Demir, Pelin Ertan, Hakan Poyrazoğlu

**Affiliations:** Gazi University, Faculty of Medicine, Department of Pediatrics, Division of Pediatric Nephrology, Ankara, Turkey; İstanbul Medipol University, Faculty of Medicine, Department of Pediatrics, Division of Pediatric Nephrology, İstanbul, Turkey; İstanbul University, Istanbul Faculty of Medicine, Department of Pediatrics, Division of Pediatric Nephrology, İstanbul, Turkey; Başkent University, Faculty of Medicine, Department of Pediatrics, Division of Pediatric Nephrology, Ankara, Turkey; Katip Çelebi University, Faculty of Medicine, Department of Pediatrics, Division of Pediatric Nephrology, İzmir, Turkey; Manisa Celal Bayar University, Faculty of Medicine, Department of Pediatrics, Division of Pediatric Nephrology, Manisa, Turkey; Erciyes University, Faculty of Medicine, Department of Pediatrics, Division of Pediatric Nephrology, Kayseri, Turkey

The 6 February 2023 earthquake that struck southern and central Turkey and northern and western Syria had unique drawbacks, such as the occurrence of two strong, destructive earthquakes nine hours apart in multiple and densely populated geographical areas, exposure to unforgiving winter conditions, and increased anxiety and fear due to multiple aftershocks [1, 2]. As of 26 March 2023, >50 000 people have been killed and many more have been injured in Turkey [3]. One recent editorial and a letter emphasized the vital importance of increased awareness of disaster preparedness and rapid action on organizational issues [4, 5]. Nongovernmental organizations including academic medical societies should take responsibility during disasters [6] and work together with other stakeholders. Since an earthquake should be considered a “kidney disaster” because of crush injuries and resultant acute kidney injury [7], the Turkish Society of Pediatric Nephrology (TSPN) took primary responsibility during the immediate and early phases of earthquake.

Search and rescue teams should include medical professionals as an integral part of the team [8] because the immediate start of appropriate medical management can change the prognosis to a large extent [7–10]. Besides protecting the lives and vital organs of earthquake victims, it is also critical to preserve skeletal integrity as much as possible and protect patients from crush injury and subsequent kidney insufficiency [11, 12]. Additionally, from a kidney point of view, children from newborn to late adolescence have unique features including different body composition, different fluid and electrolyte, and nutritional requirements, different drug dosing based on glomerular filtration rate, and different dialysis prescriptions, etc. In this regard, pediatric nephrologists are an essential part of disaster management efforts and play a very important role. Therefore, the TSPN worked coherently with other physicians including non-nephrology pediatricians, emergency care physicians, intensivists, infectious disease specialists, orthopedic surgeons, pediatric surgeons, psychiatrists, dive medicine specialists, and of course dedicated hemodialysis (HD) nurses/technicians, social workers and psychologists. Furthermore, the TSPN Council have worked coherently and complementarily with the Turkish Ministry of Health (MoH) on organizational issues. We herein summarize the collaborative efforts of the TSPN Council to overcome the shortcomings of this disaster and to maintain uninterrupted medical care. It is our belief that this experience should be publicized in order to guide all high-risk countries located in an earthquake zone in their future organization after earthquakes. Furthermore, such text is not only helpful for earthquakes but also for disasters with similar characteristics, e.g. wars.

It was critical to create a bridge between providers of care in the field taking into account their needs, and the central health authority and its healthcare delivery service in this chaotic environment. This was as important as coordinated search and rescue in the field. Disaster preparedness is a very critical issue and an absolute necessity not only for disaster victims, but also for patients with chronic diseases who require uninterrupted medical care, such as dialysis patients [13]. Therefore, the TSPN prepared an instant, rapid, multifaceted work plan to help victims and physicians working in the field (Fig. 1).

**Figure 1:**
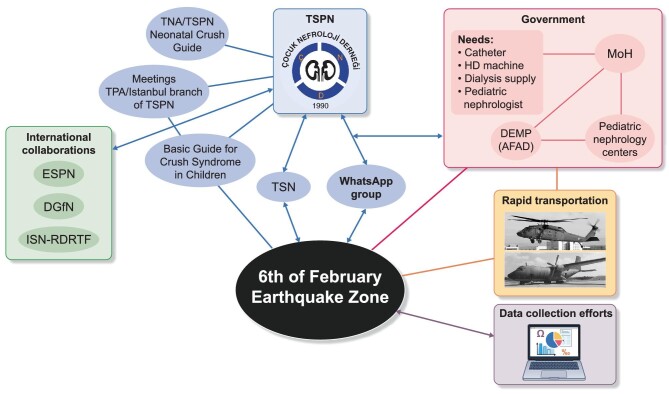
Organization of pediatric kidney care in the aftermath of the 6 February 2023 earthquake in Turkey—efforts of the Turkish Society of Pediatric Nephrology.

## RAPID COMMUNICATION WITH THE FIELD

- To coordinate activities, a Turkish Earthquake Zone WhatsApp group was established. Council members of the TSPN and pediatric nephrologists working at the hospitals in 10 affected cities were included in the group. Fortunately, our society members working at the earthquake zone were not directly affected by the earthquake. However, there were many damaged hospitals, pediatric wards and HD centers. This group was expanded to include physicians working neighboring the damaged area and finally, those at distant centers who could accept patients from the affected areas.
- With the contribution of our senior colleagues with a vast experience on disaster medicine, Prof. Dr Lale Sever (İstanbul University İstanbul Medical Faculty, Department of Pediatric Nephrology) and Prof. Dr Mehmet Şükrü Sever (İstanbul University İstanbul Medical Faculty, Department of Nephrology), the Basic Guide for Crush Syndrome in Children was adapted from the adult guide and circulated to our members on the same day [14, 15].

## DETERMINATION OF AND MEETING THE NEEDS

- Needs in terms of pediatric nephrology personnel, dialysis machines, catheters, dialysis supply and medications were determined.
- The places where a pediatric nephrologist was needed were determined.
- We created a list of volunteering pediatric nephrologists willing to offer support in the affected areas. Ten volunteers actively worked in the earthquake zone for a limited time interval of 3–5 days. Some of the pediatric nephrologists were replaced because of or to prevent burnout.

Hatay, the most deeply affected city, was visited for supporting volunteer pediatric nephrologists and to determine the needs with close contact with the field. The emergency department, dialysis unit and pediatric ward of the university hospital were investigated. Even though there was damage in different hospital buildings, the dialysis unit was intact and was actively working. The Minister of Health and Minister of Interior Relations visited the Disaster and Emergency Management Coordination Center in Hatay. The current situation was discussed and the importance of uninterrupted search and rescue efforts was emphasized to the government officials.

## COORDINATION ACTIVITIES WITH THE TURKISH AUTHORITIES

Coordination was ensured with the Disaster and Emergency Management Presidency and MoH officials in order to quickly meet the needs in terms of pediatric nephrologists. Different general directorates of the MoH were contacted to meet the different demands of pediatric nephrologists working in the field.

- General Directorate of Health Services: HD machines and dialysis supplies (catheters, filters, tubings), different consumables, medications including antibiotics, bicarbonate, calcium gluconate, and infusion fluids including albumin, were transferred, where necessary.
- General Directorate of Public Hospitals: centrally pre-set outpatient appointments were cancelled in two big city hospitals accepting too many earthquake victims in order to decrease the workload of pediatric nephrologists in these centers.
- General Directorate of Personnel: volunteer pediatric nephrologists and pediatricians were assigned to affected areas, dispensations for our colleagues who had been active in the damaged area were arranged to decrease their burden and facilitate their psychological recovery. Additionally, Turkish Psychiatry Society and Psychology Societies worked very actively in the area.

This early action facilitated precluding the coordination problem in the region in the first days of the earthquake. Personnel and material needs from the field, including dialysis consumables, which can change every moment were constantly monitored and solved quickly. Immediate transport of volunteers including pediatric nephrologists and pediatricians was arranged by the MoH and was even realized by military helicopters, where necessary. The widespread network of HD units throughout Turkey allowing rapid transfer and rescheduling prevented any interruption in HD treatments of pediatric victims.

## NATIONAL COLLABORATIONS

By cooperation with the Turkish Society of Nephrology, continuity of care was assured by contacting adult nephrologists when necessary, especially in centers without pediatric nephrologists.

Meetings were organized in collaboration with Turkish Pediatric Association and İstanbul branch of TSPN [16].

- Disasters from the perspective of pediatric nephrologist—9 February 2023
  - Crush syndrome
  - Disaster preparedness in pediatric nephrology centers
  - Study suggestions
- After the earthquake—16 February 2023
  - “How should I approach pediatric earthquake victims?”
  - “How should I protect myself as a physician?”
  - Impressions from the field

In cooperation with the Turkish Neonatology Association, a guideline was created for the follow-up of newborn babies in earthquakes [17].

## INTERNATIONAL COLLABORATIONS

- A meeting was organized by the European Society for Paediatric Nephrology (ESPN) Disaster Taskforce to help Turkish pediatric nephrologists. Free online registration support for the 55th ESPN Congress which will be held in Vilnius, Lithuania for young Turkish pediatric nephrologists who were on the front lines of the earthquake was suggested by the TSPN, and this was well accepted by ESPN council.
- Although there were enough resources in terms of dialysis equipment/supply in Turkey, the International Society of Nephrology (ISN) Renal Disaster Relief Task Force and the German Nephrology Association expressed their willingness to provide any kind of dialysis supply. Although there was no need in Turkey, these dialysis supplies were sent to Syria by the ISN Renal Disaster Relief Task Force.

## INDUSTRY

Industry, particularly the dialysis industry, helped a lot. Private dialysis center owners opened their doors for free dialysis sessions, and they used their catheters, tubing and all consumables without any restriction.

## DATA COLLECTION EFFORTS

A web-based patient database was created to collect basic patient data [18]. Ethical approval was obtained from Gazi University and the first results are being analyzed. A more detailed data collection form was also established, and ethical approval was obtained from the same university and is ready to be sent to centers.

## SHORTCOMINGS

The Turkish Society of Nephrology has kidney disaster preparedness plans but pediatricians have not been involved. This is an important shortcoming and should be considered a priority area for the pediatric nephrology community, which requires much more attention and work on it [13]. There might be also improvements needed on the side of the administration and in advance coordination with the administration. There were problems in patient transport particularly related to unequal distribution of victims among hospitals. One hospital was overloaded, another was empty despite its availability. Lastly, during the earthquake, only earthquake and geology experts, and logisticians were featured on all TV channels, newspapers and social media. Nephrologists were not consulted to obtain their perspective and suggestions for preventing kidney damage and promoting kidney care. Creating an increased awareness of the triangle of earthquake, crush syndrome and acute kidney injury among policymakers, nationally and internationally, is the responsibility of the nephrological community.

## CONCLUDING REMARKS

In this widespread tragic disaster, the TSPN was a part of the national/international collaborative effort to save patients’ lives and to decrease crush injury and resultant acute kidney injury–related morbidity. Even though the amateur effort worked nicely and the Turkish pediatric nephrology community responded well to this tragic event, it was realized once more that well-structured kidney disaster preparedness masterplans and roadmaps with algorithms for pediatric kidney disaster management are essential. Predefined disaster sister hospitals and well-structured kidney disaster action plans including the establishment of kidney disaster teams and immediate mobility of medical personnel to the affected areas should be organized. Kidney disaster management should be incorporated into the pediatric nephrology fellowship curriculum, and even the medical school curriculum. Reliable data collection during these unforeseen events and careful analysis and interpretation of the results are of particular importance for managing future events.
